# Use of the Oxford Handicap Scale at hospital discharge to predict Glasgow Outcome Scale at 6 months in patients with traumatic brain injury

**DOI:** 10.1186/1471-2288-8-72

**Published:** 2008-11-06

**Authors:** Pablo Perel, Phil Edwards, Haleema Shakur, Ian Roberts

**Affiliations:** 1 Department of Epidemiology and Population Health, London School of Hygiene & Tropical Medicine, London, UK

## Abstract

**Background:**

Traumatic brain injury (TBI) is an important cause of acquired disability. In evaluating the effectiveness of clinical interventions for TBI it is important to measure disability accurately. The Glasgow Outcome Scale (GOS) is the most widely used outcome measure in randomised controlled trials (RCTs) in TBI patients. However GOS measurement is generally collected at 6 months after discharge when loss to follow up could have occurred. The objectives of this study were to evaluate the association and predictive validity between a simple disability scale at hospital discharge, the Oxford Handicap Scale (OHS), and the GOS at 6 months among TBI patients.

**Methods:**

The study was a secondary analysis of a randomised clinical trial among TBI patients (MRC CRASH Trial). A Spearman correlation was estimated to evaluate the association between the OHS and GOS. The validity of different dichotomies of the OHS for predicting GOS at 6 months was assessed by calculating sensitivity, specificity and the C statistic. Uni and multivariate logistic regression models were fitted including OHS as explanatory variable. For each model we analysed its discrimination and calibration.

**Results:**

We found that the OHS is highly correlated with GOS at 6 months (spearman correlation 0.75) with evidence of a linear relationship between the two scales. The OHS dichotomy that separates patients with severe dependency or death showed the greatest discrimination (C statistic: 84.3). Among survivors at hospital discharge the OHS showed a very good discrimination (C statistic 0.78) and excellent calibration when used to predict GOS outcome at 6 months.

**Conclusion:**

We have shown that the OHS, a simple disability scale available at hospital discharge can predict disability accurately, according to the GOS, at 6 months. OHS could be used to improve the design and analysis of clinical trials in TBI patients and may also provide a valuable clinical tool for physicians to improve communication with patients and relatives when assessing a patient's prognosis at hospital discharge.

**Trial Registration Number:**

ISRCTN74459797

## Background

Traumatic brain injury (TBI) is an important cause of acquired disability. In evaluating the effectiveness of clinical interventions for TBI it is important to measure disability accurately. The Glasgow Outcome Scale (GOS) is the most widely used outcome measure in randomised controlled trials (RCTs) in TBI patients.[1] However, because the GOS assesses how well patients function in their daily social interactions, it is only applicable after the patient has been discharged from hospital.

Loss to follow up after hospital discharge is a common problem in clinical trials in TBI and some amount of missing data is often unavoidable.[2] If an early outcome measure was available that could predict long term disability, it could be valuable for dealing with missing data, and might potentially be used as a surrogate outcome.

The MRC CRASH Trial was a large, randomised placebo controlled trial of the effects of a 48-hour infusion of corticosteroids on death and disability, among 10,008 adults.[3] Using data from this cohort of patients we have previously identified hospital admission variables that accurately predict 6 months GOS.[4] This cohort also presents an opportunity to evaluate the predictive validity of an early disability outcome measure for TBI patients. A modified version of the Oxford Handicap Scale (OHS) was completed at hospital discharge and the GOS was completed at 6 months after injury. The OHS, which was originally developed for stroke patients, comprises six categories: no symptoms, minor symptoms, minor handicap, moderate handicap, moderately severe handicap, and severe handicap.[5] In the MRC CRASH Trial a modified form of the OHS was used in which moderate handicap and moderately severe handicap were combined. Although the OHS has been previously used in brain injury trials, its association with GOS at 6 months in TBI patients has not been previously reported.[5]

The aim of this paper is to describe the association between an early disability outcome (OHS), and a 6 months disability outcome (GOS). Specifically the objectives were to:

1) Evaluate the correlation between OHS at hospital discharge and GOS at 6 months

2) Evaluate different dichotomies of the OHS at hospital discharge in predicting GOS at 6 months

3) Evaluate the extent to which OHS at hospital discharge predicts GOS at 6 months in survivors

## Methods

### Potential predictor

The OHS (table 1) was assessed at 14 days, hospital discharge or death (whichever occur first).

**Table 1:** Original Oxford Handicap Scale and OHS used in the MRC CRASH Trial

**Original OHS**	**Modified OHS used in CRASH**
**Categories**	**Categories**

No symptoms	No symptoms

Minor symptoms that do not interfere with lifestyle	Minor symptoms

Minor handicap, symptoms that lead to some restriction in lifestyle but do not interfere with the patient's capacity to look after himself	Some restriction in lifestyle but independent

Moderate handicap, symptoms that significantly restrict lifestyle and prevent totally independent existence	Dependent but not requiring constant attention

Moderately severe handicap, symptoms that clearly prevent independent existence though not needing constant attention	

Severe handicap, totally dependent patient requiring constant attention night and day	Fully dependent requiring attention day and night

	Death

Variables that have previously been reported to be associated with the outcome were considered as potential confounders and included in an adjusted model: age, Glasgow Coma Scale (GCS) at randomization, pupil reactivity, whether the patient sustained a major extra cranial injury and computerised tomography (CT) scan results.[4]

### Outcome

The outcome was GOS at 6 months. The GOS comprises five categories: death, persistent vegetative state, severe disability, moderate disability and good recovery.[6] GOS was dichotomised for analysis in the CRASH Trial into favourable outcome (good recovery or moderate disability) and unfavourable outcome (severe disability, persistent vegetative state or death). We created two further dichotomies: good recovery versus other outcomes, and survival versus death.

### The sample of patients

The MRC CRASH trial was a large international double-blind randomised placebo-controlled trial of the effect of early administration of a 48-h infusion of a corticosteroid (methylprednisolone) on the risk of death and disability after TBI. The characteristics of the patients randomised, and results of the trial have already been reported in detail.[3,7] Briefly, adults (aged 16 years or older) with a head injury and a GCS of 14 or less were randomly allocated to commence either a 48 hour infusion of methylprednisolone or matching placebo within eight hours of injury; patients from 239 hospitals in 48 countries were randomised. All collaborating MRC CRASH investigators were required to secure local ethics or research committee approval before recruitment could begin. Patients with clinically significant head injury are unable to give valid informed consent. Local ethics committees set consent procedures for participating hospitals. Some allowed consent waiver and others consent from a legal representative. We always adhered to these requirements.

**Figure 1:**
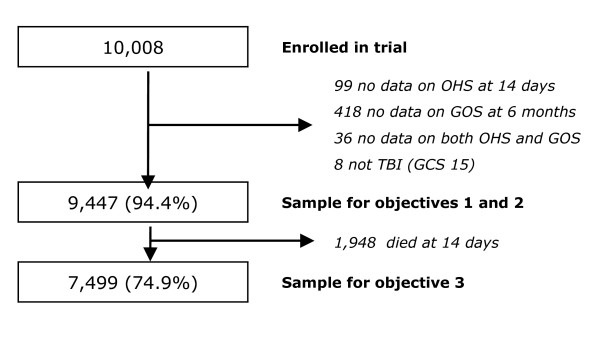
Flowchart of patients used in the analysis.

Of 10,008 study participants enrolled in the MRC CRASH Trial, 99 (1%) had missing data on the OHS, 418 (4.2%) had missing data on the GOS at 6 months, and 36 (0.3%) had missing data for both OHS and GOS. A further 8 patients were excluded from analysis as they had a Glasgow Coma Scale (GCS) score of 15 at randomisation. Analysis for objectives 1 and 2 were therefore performed using data for 9,447 (94.4%) patients (figure 1). For the third objective (predictive validity of OHS among survivors), the 1,948 patients who died within 14 days of admission were excluded and the analysis was based on data for the remaining 7,499 patients (figure 1).

### Analysis

#### Objective 1

A cross-tabulation between the OHS and GOS categories was performed. Their relation was assessed with the Spearman rank correlation index.

#### Objective 2

The validity of the different dichotomies of the OHS for predicting GOS at 6 months was assessed by calculating sensitivity, specificity and the c statistic (an equivalent concept to area under the receiver operator characteristic curve).

#### Objective 3

A logistic regression model was first fitted including only OHS as explanatory variables (model 1). A second model was then fitted that also included demographic and clinical variables (model 2). Finally, a third model was fitted that included all variables in model 2, plus CT scan variables. All the demographic, clinical and CT variables have been previously reported as being independently associated with unfavourable outcome at 6 months.[4] For each model we analysed its discrimination using the c statistic and calibration (graphically and with the Hosmer-Lemeshow test).

We then estimated the positive predictive value (with 95% confidence intervals) of each OHS category for GOS at 6 months.

## Results

### General characteristics of the population

**Figure 2:**
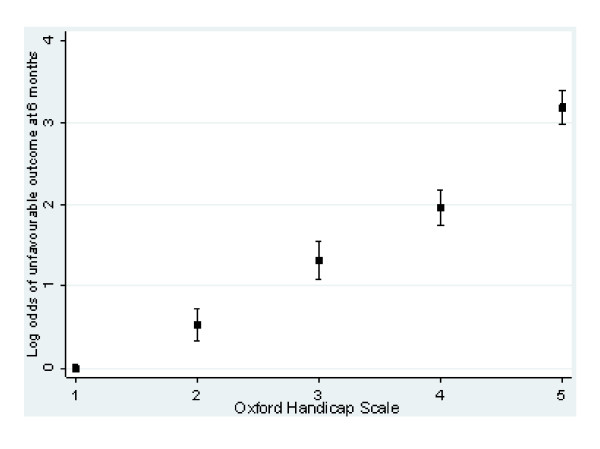
Relationship between Oxford Handicap Scale and unfavourable outcome (GOS) at 6 months.

Table 2 shows the characteristics of the sample included in the analysis. At 14 days 1,863 (19%) were dependent and 1,948 patients had died (21%). At 6 months, 3,525 (37.3%) patients were severely disabled or had died. Most deaths (84%) occurred within the first 14 days. OHS at 14 days and GOS at 6 months were highly correlated (Spearman rank correlation coefficient 0.75) and they showed a linear relationship (figure 2).

**Table 2:** Glasgow Outcome Scale at 6 months by Oxford Handicap Scale at 14 days

	**Glasgow Outcome Scale at 6 months**								
**Oxford Handicap Scale at 14 days**	***Good recovery***		*Moderate disability*		***Severe disability***		*Death*		***Total***

	**n**	**%**	n	%	**n**	**%**	n	%	**n**

*No symptoms*	1,910	79	334	14	150	6	17	1	2411

*Minor symptoms*	1,646	67	537	22	233	9	42	2	2,458

*Some restriction in lifestyle but independent*	354	46	246	32	147	19	20	3	767

*Dependent but not requiring constant attention*	232	30	273	35	221	29	45	6	771

*Fully dependent requiring attention day & night*	148	14	242	22	457	42	245	22	1,092

*Death*	*0*	*0*	*0*	*0*	*0*	*0*	*1948*	*100*	*1948*

*Total*	4,290	45	1,632	17	1,208	13	2317	25	9,477

### OHS for predicting 6 months outcome

Five dichotomies of the OHS were considered (Table 3).

**Table 3:** Dichotomies of OHS for determining unfavourable outcome

	*A*	*B*	*C*	*D*	*E*
*No Symptoms*	**No**	**No**	**No**	**No**	**No**

*Minor Symptoms*	Yes	**No**	**No**	**No**	**No**

*Some restriction in lifestyle but independent*	Yes	Yes	**No**	**No**	**No**

*Dependent but not requiring constant attention*	Yes	Yes	Yes	**No**	**No**

*Fully dependent requiring attention day and night*	Yes	Yes	Yes	Yes	**No**

*Death*	Yes	Yes	Yes	Yes	Yes

When their validity was assessed in relation to unfavourable outcome as defined by the GOS (severe disability or death), dichotomy D showed the highest discrimination (c statistic) with high specificity (Table 4).

**Table 4:** Validity of the Oxford Handicap Scale at 14 days for Glasgow Outcome Scale at 6 months

OHS dichotomy	Sensitivity	Sensitivity	C stat
A	95.3	37.9	66.6

B	87.5	74.8	81.1

C	82.7	84.9	83.8

**D**	**75.2**	**93.4**	**84.3**

E	55.3	100.0	77.6

**Figure 3:**
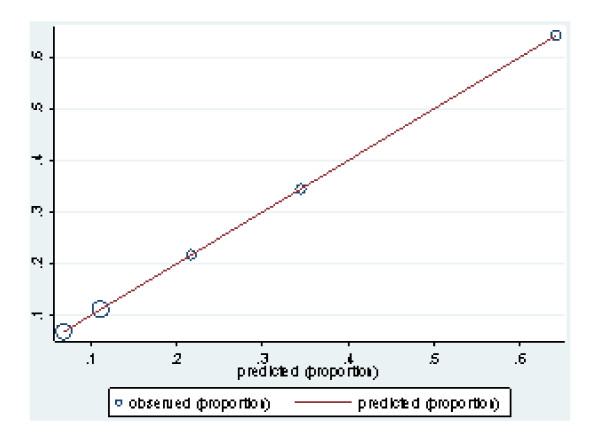
Calibration of model 1.

Among survivors at hospital discharge the OHS showed a strong association with GOS at 6 months. The crude analysis showed that patients who were fully dependent at 14 days had 24 higher odds of an unfavourable outcome at 6 months. Although adjusting for known prognostic factors attenuated the strength of the association, OHS remained a strong predictor with a highly statistically significant test (Table 5). Most importantly, when considered alone, OHS showed very good discrimination and excellent calibration (H-L = 1) (figure 3).

**Table 5:** Association between OHS and unfavourable outcome (GOS) among survivors

	***Model 1***			*Model 2*			***Model 3***		
		**95% CI**			95% CI			**95% CI**	
						
OHS	**OR**	**Lower**	**Upper**	OR	Lower	Upper	**OR**	**Lower**	**Upper**

				1.0			1.0		

Minor symptoms	1.7	1.4	2.1	1.6	1.3	1.9	1.6	1.3	2.0

Some restriction in lifestyle but independent	3.7	3.0	4.7	2.7	2.1	3.4	2.7	2.1	3.5

Dependent but not requiring constant attention	7.1	5.7	8.8	4.5	3.6	5.7	4.7	3.7	6.0

Fully dependent requiring attention day & night	24.1	19.8	29.4	12.9	10.3	16.2	13.3	10.4	16.9

*C statistic for the model*	0.78			0.83			0.83		

Model 1: OHS

Model 2: model 1 plus GCS, pupil reactivity, major extra-cranial injury and age

Model 3: model 2 plus CT findings (petechial haemorrhages, obliteration of the third ventricle or basal cisterns, subarachnoid bleed, midline shift, non evacuated haematoma)

Table 6 shows the prediction of different 6 months outcomes (as measured with GOS) according to the hospital discharge OHS. For example, a patient with minor symptoms at hospital discharge will have a probability of approximately 67% of good recovery, 89% of good recovery or moderate disability and 98% of survival at 6 months.

**Table 6:** Prediction of three dichotomies of GOS at 6 months according to OHS

	*Good recovery*			*Good recovery or Moderate disability*			*Survival*		
		95% CI			95% CI			95% CI	
						
OHS	**%**	Lower	Upper	%	Lower	Upper	%	Lower	Upper

No symptoms	79.2	77.6	80.8	93.1	92.1	94.1	99.3	99.0	99.6

**Minor symptoms**	**67.0**	65.1	68.8	**88.8**	87.6	90.1	**98.3**	97.8	98.8

Some restriction in lifestyle but independent	46.1	42.6	49.7	78.2	75.3	81.2	97.4	96.3	98.6

Dependent but not requiring constant attention	30.1	26.8	33.3	65.5	62.1	68.9	94.2	92.5	95.8

Fully dependent requiring attention day & night	13.6	11.5	15.6	35.8	32.9	38.6	77.6	75.1	80.0

## Discussion

### Principal findings

We found that the OHS is highly correlated with GOS at 6 months with evidence of a linear relationship between the two scales. The OHS dichotomy that separate patients who were severely dependent or dead (dichotomy D) showed the greatest discrimination. Among survivors at hospital discharge the OHS showed a very good discrimination and excellent calibration when used to predict GOS outcome at 6 months.

### Strengths and weakness of the study

To our knowledge this is the first study that evaluated the predictive validity of a simple scale for disability at hospital discharge in TBI patients. The main strengths of our study include the large sample size which ensures precision in our estimates, and the inclusion of patients from both high and low & middle income countries, which increases the generalizability of our conclusions. The main limitation is that the measurement of OHS was not standardized between centres. However, because we would expect that any measurement error would result in non-differential misclassification, in general we would expect that the association reported would be underestimated rather than overestimated. Finally, our study is the first to report this association which should therefore be examined in an external cohort of patients in order to confirm the findings.

### Comparison with other studies

The incidence of unfavourable GOS outcome at 6 months in our cohort was lower in comparison to one reported in a series of TBI cohorts.[8] However, unlike ours, most of these cohorts had been restricted to severe TBI patients. The OHS has previously been used in RCTs of brain injury patients, and Bamford et al. reported good inter-observer agreement (a weighted kappa of 0.72).[5] Ours is the first study in TBI which has evaluated the relationship between OHS and GOS. Nevertheless, previous studies have shown a good agreement between the Modified Rankin Scale (the scale from which the OHS was derived) and the GOS.[9]

## Conclusion

We have shown that OHS is strongly related and predicts accurately the GOS at 6 months. It may therefore be helpful in tackling the problem of missing data in clinical trials in TBI. It might also serve as a potential surrogate outcome measure and this application should be explored in further studies. If our findings are replicated, OHS could be a simple and useful outcome measure to use in trials in settings for which long term follow-up is problematic. Furthermore, OHS could be a useful variable to collect in rehabilitation trials in TBI patients to ensure that there is a similar distribution of disability among participants between groups at baseline. We have also shown that, among survivors, the OHS is able to predict disability at 6 months and thus may provide a valuable clinical tool for physicians to improve communication with patients and relatives when assessing a patient's prognosis at hospital discharge.

## Competing interests

The authors declare that they have no competing interests.

## Authors' contributions

PP designed the study, performed the analysis and prepared the manuscript. IR conceived the study, revised and drafted the manuscript. PE and HS revised and drafted the manuscript.

## Pre-publication history

The pre-publication history for this paper can be accessed here:


